# Risk factors for impaired health-related quality of life in a cohort of pediatric patients with inborn metabolic diseases

**DOI:** 10.1007/s00431-021-04300-y

**Published:** 2021-10-31

**Authors:** Sandy Siegert, Anne Roscher, Dorothea Moeslinger, Vassiliki Konstantopoulou, Marion Herle

**Affiliations:** Department of Pediatrics and Adolescent Medicine, Medical University of Vienna, Währinger Gürtel 18-20, 1090 Vienna, Austria

**Keywords:** Health-related quality of life, Inborn metabolic diseases, Inborn errors of metabolism, Risk factors, Children

## Abstract

In the last decade, health-related quality of life (HrQoL) has become an increasingly important outcome parameter in children and adolescents with chronic health conditions; among them are pediatric patients with inborn metabolic diseases (IMDs). Hence, knowledge on this topic is increasing, but findings on non-medical influences on the HrQoL of IMD patients are still scarce. In the present study, we retrospectively evaluated the self-reported generic HrQoL of a cohort of pediatric patients (ages 7 to 17 years) with diverse IMDs (*n* = 204) and explored associations between HrQoL and psychosocial and medical characteristics of the patients. We aimed to identify risk factors for impaired HrQoL to improve and tailor support for the patients and economize resources. Generic HrQoL was assessed with the KINDL-R questionnaire. We compared the HrQoL scores to published German normative data and analyzed the impact of demographic variables and intellectual and psychosocial functioning on the HrQoL. Moreover, we examined the influence of the diagnostic category and the health impairment (as judged by the physicians) on our patients’ HrQoL. Overall, the HrQoL of the adolescent patients was comparable to the HrQoL of the norm group. Disorders of intellectual development, impaired psychosocial functioning, and a severe health impairment were associated with lower HrQoL scores.

*Conclusion*: We recommend evaluating these factors in children and adolescents with IMDs to identify patients at risk for impaired HrQoL.

**Table:** 

**What is Known:** *• Studies on HrQoL in pediatric patients with IMDs mainly focused on subgroups with specific diagnoses and found normal HrQoL in some of those subgroups.* *• In healthy children and adolescents as well as in pediatric patients with various chronic diseases, associations between psychosocial factors and HrQoL are well known.*	
**What is New:** *• Impaired psychosocial functioning, disorders of intellectual development, and a significant disease and/or treatment burden are risk factors for impaired HrQoL in pediatric patients with IMDs.* *• Evaluating these factors in children and adolescents with IMDs can help identify patients and families in need of enhanced psychological support.*

**What is Known:**

*• Studies on HrQoL in pediatric patients with IMDs mainly focused on subgroups with specific diagnoses and found normal HrQoL in some of those subgroups.*

*• In healthy children and adolescents as well as in pediatric patients with various chronic diseases, associations between psychosocial factors and HrQoL are well known.*

**What is New:**

*• Impaired psychosocial functioning, disorders of intellectual development, and a significant disease and/or treatment burden are risk factors for impaired HrQoL in pediatric patients with IMDs.*

*• Evaluating these factors in children and adolescents with IMDs can help identify patients and families in need of enhanced psychological support.*

## Introduction

Inborn metabolic diseases (IMDs) represent a large, heterogeneous group of genetic disorders that lead to a dysfunctional metabolism with observable effects already in early childhood. A classification of inherited metabolic disorders is provided by the Society for the Study of Inborn Errors of Metabolism (SSIEM) [1]. Due to these diseases’ heterogeneity, patients exhibit mild to severe symptoms and are faced with different and sometimes challenging therapeutic options. Despite therapy, some of these conditions still lead to developmental disturbances, disorders of intellectual development, organ damage, physical impairment, and a shortened life expectancy (e.g., [2, 3]).

Psychological developmental assessment and health-related quality of life (HrQoL) measurement are recommended in several guidelines for the professional care of different IMDs (e.g., [4–8]). The term HrQoL reflects an individual’s or a group’s perceived impact of the disease and its treatment on their physical and mental health over time. Questionnaires measuring HrQoL provide a powerful tool to evaluate psychosocial stressors in the patients’ and their families’ lives [9]. Knowledge about HrQoL in children with IMDs is increasing, but findings are inconsistent. Previous studies have mainly investigated subgroups of these patients and the influence of treatment protocols on their HrQoL (e.g., [2, 10–14]).

In a recent study, Cano et al. found normal self-reported HrQoL in a large cohort of children suffering from intoxication-type IMDs [11]. In contrast, Bösch et al. described a significantly lower self-reported HrQoL in pediatric patients with IMDs of the intoxication type than in healthy children [12]. Interestingly, they found that the emotional scale score of patients with phenylketonuria (PKU) was lower compared to patients with IMDs causing acute exacerbations and life-threatening metabolic crises. This finding highlights the necessity to consider non-medical factors modulating HrQoL in these patients. However, studies on psychosocial influences on HrQoL of patients with IMDs are scarce [15]. Jamiolkowski et al. reported no correlation between HrQoL and behavioral or emotional problems in patients with organic acidurias and urea cycle disorders [16].

Our study investigated the HrQoL of pediatric patients with diverse IMDs receiving treatment at a pediatric metabolic center and aimed to understand associations between the patients’ medical and psychosocial characteristics and their self-reported HrQoL to identify risk factors for impaired HrQoL. The findings could improve the psychosocial support for these patients and their families by economizing resources and individualizing support.

## Methods

### Data collection

This single-center retrospective study was conducted at the center of inherited metabolic diseases in Vienna, Austria, after ethical approval (Ethics Committee of the Medical University of Vienna, Number 1474/2018). Data was collected between 2002 and 2018 as part of the routine examination of children and adolescents with IMDs. The standardized routine examination protocol for these patients intends to provide regular psychological assessments at certain ages and defined time points, independent of clinical symptoms. It contains a specific set of questionnaires and psychological tests [17]. The results of the assessment are documented in the patient’s medical record. For this study, we extracted the data from the medical records and included data of children from the age of seven.

### Participants

The study sample consists of children and adolescents with a confirmed diagnosis of an IMD who are receiving or received treatment at the pediatric center of inherited metabolic diseases in Vienna and participated in the routine psychological assessment between 2002 and 2018. We excluded patients who did not complete the HrQoL questionnaire.

### Measures

#### HrQoL

To evaluate the generic HrQoL, the German version of the standardized questionnaire KINDL-R [18] was administered. The questionnaire is available in several languages, and its psychometric properties have been tested in various studies (e.g., [19]). It contains age-appropriate versions for the patient’s self-report and parent proxy reports. We evaluated data from the self-report versions KINDL Kid (7–12 years) and KINDL Kiddo (13–17 years). Each version consists of 24 items yielding a total HrQoL score and six subscales: physical well-being, emotional well-being, self-esteem, family, friends, and school. Higher scores represent better HrQoL. The maximum score is 100 for all subscales and the total score.

#### Demographic data and diagnosis

The patients’ demographic and medical data were directly gathered from their medical files. Diagnoses were assigned to the diagnostic categories according to the SSIEM classification [1]. Infrequent diagnosis groups with less than ten patients were combined into a sixth category, “others.”

#### Expert rating on patient’s health impairment

To classify the patient’s impairment in daily life due to the disease or its treatment, we established the new factor “health impairment.” Three physicians who are experts in IMDs and were involved in treating the patients independently assigned the patients to one of three categories according to their impairment: (1) no impairment, (2) mild-moderate impairment in daily life (e.g., receiving protein-restricted diet, daily oral medication), and (3) severe impairment in everyday life (complex treatment, frequent stays in hospital, reduced life expectancy). In 90 patients (44.1%), the rating of at least two experts matched. These patients were subsequently allocated to the respective impairment grade by the first author, who did not know the patients. Ninety-four patients (46.1%) were known to only one physician and could not be assigned to a category.

#### Intellectual functioning

Assessment of intellectual functioning was performed with the Kaufman Assessment Battery for Children (K-ABC or K-ABC II, German version [20, 21]) or the Adaptive Intelligence Diagnosticum 2 (AID2 [22]). For this study, only the respective global test score (mental processing index for K-ABC/K-ABC II and IQ for AID 2) was used. It was divided into four categories: (a) above average: > 115, (b) average: 85–115, (c) below average: 70–84, (d) cognitive impairment: < 70.

#### Psychosocial functioning

To screen for emotional and behavioral problems, parents and patients aged ≥ 11 years filled in the German version of the Strengths and Difficulties Questionnaire (SDQ) [23]. Both the self-report version (11–17 years) and the parent proxy version (4–17 years) of this behavioral screening questionnaire consist of 25 items divided between 5 subscales. Scores can be classified as normal, borderline, or abnormal. For this study, the total score was used. Parent proxy and self-report (if applicable) total scores in the normal range indicated a patient with no psychosocial functioning issues. Self-report, parent proxy, or both total scores above the average indicated “abnormal” functioning.

### Data analysis

Descriptive statistics and ANOVAs were performed with the software SPSS (IBM SPSS Statistics 25.0). To evaluate the patients’ self-reported HrQoL, we compared an age-matched group (11–17 years, *n* = 102) of our sample to the published normative data from a large epidemiological study from Germany (Bella Study [9]). The Student *t*-test was used for the statistical comparison.

For each of the following variables, ANOVAs were performed to explore their influence on total HrQoL and subscale scores: (a) age group (two groups: 7–12 years vs. 13–17 years) and gender (two groups: female/male), (b) diagnostic category (six groups: amino acid and peptide metabolism disorders, carbohydrate metabolism disorders, fatty acid and ketone body metabolism defects, lysosomal disorders, disorders of vitamins and (non-protein) cofactors, others), (c) health impairment (three groups: none or almost none, mild to moderate, severe impairment), (d) intellectual functioning (four groups: above average, average, below average, cognitive impairment). For all analyses, HrQoL subscale scores and the HrQoL total score were entered as dependent variables. For subscale analyses, significance levels were calculated by applying Bonferroni-Holm correction to the *p*-values. Furthermore, we used the Bonferroni-Holm correction for the post hoc analyses of significant interaction effects. We applied the Student *t*-test to explore the influence of (e) family structure (two groups: living together with both parents versus other) and (f) psychosocial functioning (total SDQ score, two groups: normal versus abnormal). *P*-values ≤ 0.05 were considered statistically significant. Effect sizes were calculated for significant results of post hoc analyses (Cohen’s *d*), *t*-tests (Cohen’s *d*), and ANOVA (partial eta squared, *η*^2^_Part_). Generally, Cohen’s *d* effect sizes > 0.2 are considered small effects, > 0.5 medium effects, and > 0.8 large effects. Accordingly, for partial eta squared, Cohen suggested borders for small (*η*^2^ ≥ 0.01), medium (*η*^2^ ≥ 0.06), and large (*η*^2^ ≥ 0.14) effect sizes [24].

## Results

### Sample characteristics

In 235 out of 249 (94.3%) psychological assessments, the child or adolescent filled in a KINDL questionnaire. From 31 patients, two datasets were available, as they had been assessed twice, once in childhood and once in adolescence. To avoid the bias of repeated measures, we chose only one of those datasets for evaluation. We alternated in including either the first or second assessment data. Hence, we analyzed data sets from 204 patients (118 children, 86 adolescents). Descriptive data on the study sample are summarized in Table 1.

**Table 1:** Patients’ characteristics (*N* = 204)

**Age at assessment**	
Mean	11.89 years
Standard deviation	2.34 years
Range	7.00–17.33 years
**Age group**	Total number of patients (in percentage)
Children (7–12 years)	118 (57.8%)
Adolescents (13–17 years)	86 (42.2%)
**Gender**	
Male	109 (53.4%)
Female	95 (46.6%)
**Family type**	
Both parents	167 (81.9%)
Other	37 (18.1%)
**Diagnostic category**	
Amino acid and peptide metabolism disorders	117 (57.4%)
Carbohydrate metabolism disorders	35 (17.2%)
Fatty acid and ketone body metabolism defects	12 (5.9%)
Lysosomal disorders	19 (9.3%)
Disorders of vitamins and (non-protein) cofactors	15 (7.4%)
Others	6 (2.9%)
**Health impairment (Patient's impairment in daily life)**	
None	16 (7.80%)
Mild/moderate	50 (24.5%)
Severe	24 (11.8%)
No confirmed rating	114 (56.0%)
**Intellectual functioning (IQ)**	
Above average (≥ 115)	21 (10.3%)
Average (85–114)	118 (57.8%)
Below average (70–84)	41 (20.1%)
Disorder of intellectual development (≤ 69)	18 (8.8%)
No assessment	6 (2.9%)
**Psychosocial adjustment**	
Normal	155 (76.0%)
Abnormal	44 (21.6%)
Unknown	5 (2.5%)

Patients’ mean age and frequency distributions of the assessed factors

### Comparison to normative data

**Fig. 1:**
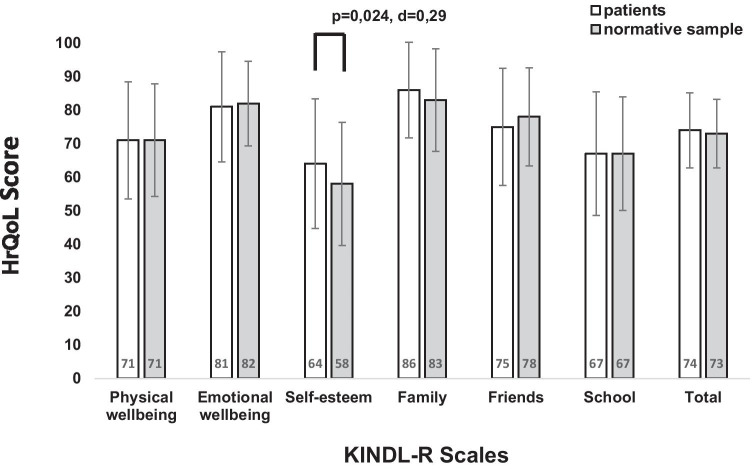
Comparison of the age-matched subgroup of patients with German normative data (mean subscale-scores, standard deviation, and total HrQoL score). The significant group difference is marked with *p*-value and Cohen’s *d*

The mean KINDL-R total score of the sample was 74.1 ± 11.2. A *t*-test revealed no statistical difference in total HrQoL between the normative sample (total HrQoL = 73.0 ± 10.2) and our age-matched subgroup of patients (total HrQoL = 74.0 ± 11.2; Fig. 1). Our patients, however, reached higher HrQoL scores in the subscale “self-esteem” (63.8 ± 19.3) compared to the normative sample (score = 58.4 ± 18.3, *t*(1994) = 2.88, *p* = 0.02, Cohen’s *d* = 0.29; Fig. 1).

### Associations between psychosocial and medical characteristics of the patients and HrQoL

Table 2 shows significant results of the statistical analyses on HrQoL modulating factors.

**Table 2:** Overview of subscales with significant group differences in HrQoL scores

HrQoL scale		Physical well-being Mean	Emotional well-being Mean	Self-esteem Mean	Family Mean	Friends Mean	School Mean	Total HrQoL Mean
Total (*N* = 204)		72.9	80.8	63.6	82.3	75.9	68.9	74.1
Age groups	Children (7–12 years)				*79.1*			
	Adolescents (13–17 years)				**86.7**			
Health impairment (expert rating)	No impairment	**74.6**			*68.0*			
	Mild-moderate	**79.2**			**85.1**			**76.1**
	Severe	*60.7*			*74.0*			*66.8*
Intellectual functioning	Above average	**77.6**						
	Average	**75.1**						**75.8**
	Below average							
	Disorder of intellectual development	*60.4*						*66.9*
Psychosocial functioning	Normal	**75.4**	**84.1**		**85.3**	**78.6**	**71.8**	**76.6**
	Abnormal	*63.7*	*70.9*		*72.6*	*67.3*	*59.2*	*65.8*

HrQoL modulating factors. Significant hrQoL scores as per ANOVA and post hoc tests with *p*-values < 0.05 are highlighted: bold, higher HrQoL scores; italic, lower HrQoL scores. Missing values represent non-significant results

#### Age and gender

Analysis of the distribution of male and female patients did not reveal any significant difference (*X*^2^(2) = 0.70, *p* = 0.40). The two-way ANOVA showed a significant main effect of “age group” on the subscale “family” with a higher score in adolescents (86.7 ± 14.1) compared to children (79.1 ± 17.8, *F*(1) = 9.95, *p* = 0.01, *η*^2^_Part_ = 0.05). We did not find any main effects or interaction effects on the total HrQoL score or the remaining subscales.

#### Family structure

We did not find any differences in HrQoL between children who live with their parents and children from single-parent families, stepfamilies, or foster families (family structure “other”).

#### Diagnostic category and health impairment

The ANOVA with the factor “diagnostic category” revealed no significant differences in HrQoL between the various diagnostic categories. The ANOVA with the factor “health impairment” revealed significant main effects on the total HrQoL score (*F*(2) = 5.70, *p* = 0.01) and the subscales “physical well-being” (*F*(2) = 9.90, *p* < 0.001] and “family” (*F*(2) = 6.98, *p* = 0.01). Post hoc results for the total HrQoL score confirmed a significantly lower score in patients with severe impairment (total HrQoL score = 66.8 ± 12.2) compared to patients with mild to moderate impairment (total HrQoL score = 76.1 ± 11.3, *p* = 0.01, Cohen’s *d* = 0.79). Moreover, post hoc tests showed that patients with severe impairment scored significantly lower on the subscale “physical well-being” (60.7 ± 18.0) compared to patients with mild to moderate impairment (score = 79.2 ± 15.6, *p* < 0.001, Cohen’s *d* = 1.10) and patients with no impairment (score = 74.6 ± 18.2, *p* = 0.04, Cohen’s *d* = 0.77). As for the subscale “family,” patients with mild to moderate impairment showed significantly higher scores (85.1 ± 13.2) than patients with no impairment (score = 68.0 ± 21.9, *p* = 0.02, Cohen’s *d* = 0.95) and patients with severe impairment (score = 74.0 ± 22.7, *p* = 0.02, Cohen’s *d* = 0.70). A chi-square test ruled out distributional effects between age-group and impairment grade (*X*^2^(1) = 0.09, *p* = 0.96).

#### Intellectual functioning

ANOVA on HrQoL with the factor “intellectual functioning” (four groups) indicated a significant influence (*F*(3) = 3.66, *p* = 0.01) on the total HrQoL. Post hoc testing confirmed lower total HrQoL scores in patients with disorders of intellectual development (total HrQoL = 66.9 ± 8.5) compared to patients with average intellectual abilities (total HrQoL = 75.8 ± 10.9, *p* = 0.004, Cohen’s *d* = 0.84). Moreover, ANOVA revealed a significant main effect of the factor “intellectual functioning” on the subscale “physical well-being” (*F*(3) = 4.48, *p* = 0.03). Post hoc analysis revealed lower “physical well-being” scores in patients with disorders of intellectual development (score = 60.4 ± 13.7) compared to patients with average intellectual abilities (score = 75.1 ± 17.3, *p* = 0.003, Cohen’s *d* = 0.87) and patients with higher-than-average intellectual abilities (score = 77.6 ± 12.8, *p* < 0.001, Cohen’s *d* = 1.31). A chi-square test ruled out distributional effects of intellectual functioning and impairment grade (*X*^2^(6) = 6.91, *p* = 0.33).

#### Psychosocial functioning

SDQ self-report and parent proxy-report data of 199 patients were collected. In 155 patients (76%), the total difficulties score was normal. Patients with an abnormal total difficulties score showed significantly lower total HrQoL scores (65.8 ± 10.1) compared to patients with a normal total difficulties score (total HrQoL score = 76.6 ± 10.2, *t*(196) = 6.16, *p* < 0.001, Cohen’s *d* = 1.08). HrQoL scores of patients with an abnormal total difficulty score remained consistently lower in all subscales (except “self-esteem”) with all *p*-values below 0.01.

## Discussion

In this study, we investigated the self-reported generic HrQoL and its associations with psychosocial and medical characteristics in a cohort of over 200 children and adolescents affected by an IMD and receiving special care in a large, acknowledged center for pediatric IMDs. Interestingly, our adolescent patients’ HrQoL was comparable to a normative sample from a German epidemiological study [9]. This finding is in line with a recent report on normal self-reported HrQoL in a subgroup of French pediatric IMD patients [11].

Moreover, these results are compatible with several HrQoL studies in children with congenital or early infancy chronic diseases showing a normal self-reported HrQoL [25]. It is assumed that a normal HrQoL indicates an adaptation to the burden of disease and the development of coping strategies, especially in non-progressive conditions [25, 26]. Thimm et al. assumed that living with an IMD might entail valuable aspects such as more supportive parental care or feelings of satisfaction about managing treatment requirements [27]. Interestingly, we found even higher HrQoL scores for the subscale “self-esteem” in our cohort, corroborating this assumption. Importantly, Cano et al. who used a French questionnaire and French normative data, report higher than average HrQoL scores in the older subgroup of patients (13–17 years) and lower HrQoL scores in the younger subgroup of children (aged 8–12 years) [11].

In contrast to our results, Bösch et al. who adopted the PedsQL to measure HrQoL in a slightly younger cohort (average age 11 years), found significantly lower scores compared to normative data from healthy American children [12]. Since our comparison was limited to patients aged 11–17 (average age 14 years), findings on average and above-average HrQoL scores might apply to older children and adolescents who have already developed coping strategies and learned how to co-manage their treatments. However, our finding of normal HrQoL in adolescents with IMDs should be interpreted with caution due to the diseases’ heterogeneity. It is probably not applicable to adolescents with severe disease-related burdens. Furthermore, additional analyses of our dataset revealed that the result of a higher self-esteem in the adolescent patient group is especially true for patients with normal cognitive and psychosocial functioning (data not shown).

Notably, scores of the subscale “family” were lower in children than in adolescents. Cano et al. reported similar findings in children suffering from intoxication type IMDs [11]. Adolescents might become aware that they depend more on their caregivers than healthy peers and avoid conflicts within the household. Moreover, as adolescents were conscious that the questionnaire was not anonymous, the social-desirability bias could have interfered with the interpretation of HrQoL scores.

Several studies on chronically ill and healthy children showed that gender influences HrQoL [9, 28]. Interestingly, we did not find gender effects in our data, suggesting that other factors have a more robust influence on our cohort.

The diagnostic category itself did not significantly modulate the HrQoL. This finding may be due to the variability within diagnostic categories regarding the influence of the various diseases and treatments on the patients’ daily lives. The health impairment grade, however, influenced the self-reported HrQoL of our patients. A higher grade of health impairment was associated with a lower HrQoL, especially on the subscales “physical well-being” and “family.” We assume that significant disease-related impairments impose challenges and limitations that protective factors cannot mitigate. Furthermore, Bilginsoy et al. showed that controlling the effects of the child’s illness on their social life represents a source of stress for parents of children suffering from PKU [29]. Hence, the perception of parental stress might have influenced the family HrQoL of our patients.

On the other hand, patients without health impairment also showed lower HrQoL scores on the subscale “family” than mildly impaired patients. This finding supports the assumption that children without impairment tend to have more conflicts with their parents and underline the importance of the family system for patients bearing the burden of their diagnosis. Hence, evaluating the patient’s health impairment helps identify patients and families needing enhanced psychosocial support. However, to best support the children and their families, it would be important to also consider the parents’ proxy reports as well as the parental HrQoL.

Furthermore, we found a significant association between HrQoL and intellectual functioning; patients with disorders of intellectual development showed lower total HrQoL and physical well-being scores than children with average or higher intellectual abilities. As intellectual functioning levels were equally distributed among health impairment categories, a higher impairment grade in patients with disorders of intellectual development does not explain this result. We assume that lower cognitive capacities prevent patients from developing optimal strategies to cope with the limitations resulting from their disease thus indicating the need for support in establishing adequate coping strategies. As expected, patients with lower psychosocial functioning reported a lower HrQoL in almost all KINDL scales. Similar findings were reported for children with propionic academia [2], chronically ill children in general [28], and healthy children [9]. In a longitudinal study, Otto et al. showed that mental health problems are a risk factor for diminished HrQoL in children and should be addressed in prevention programs [30].

## Limitations

Although large, our sample reflects the frequency distribution of different IMDs in our metabolic center and may not be representative. Disease-specific effects of more frequent diagnostic groups of IMDs might be overrepresented. Moreover, the KINDL-R is a generic questionnaire that is less sensitive to disease- or treatment-specific limitations of HrQoL than disease-specific questionnaires [16]. Applying disease-specific HrQoL questionnaires could have possibly revealed different findings. Furthermore, comparison with normative data was restricted to children older than ten years. Although we excluded patients with evident and severe difficulties in verbal comprehension, patients with impaired cognitive functioning might have had difficulties in understanding and answering the KINDL questionnaire. Such difficulties could have had an influence on their HrQoL scores. Furthermore, the patients knew that the results of their psychological assessments would be discussed with their parents. Thus, social desirability might have inflated scores.

## Conclusion

We found that, on average, adolescents suffering from IMDs can achieve a normal HrQoL. Our study revealed three essential factors that affect the self-reported HrQoL of pediatric IMD patients highlighting the need for distinct psychological support for specific subgroups.

(I) Health impairment: The care team needs to judge the impairment due to the disease or treatment and offer psychological support to highly impaired patients. As family HrQoL is substantially affected by health impairment, family counseling and therapy are recommended.
(II) Cognitive functioning: Patients with disorders of intellectual development should be identified and offered support in establishing coping strategies.
(III) Psychosocial functioning: Screening for mental health problems in IMD patients with a brief questionnaire addressing behavioral and emotional problems is essential. Early treatment of mental health problems could positively impact the patients’ HrQoL.

## Data Availability

All data are available on request.

## Abbreviations

AID 2: Adaptive Intelligence Diagnosticum 2
ANOVA: Analysis of variance
HrQoL: Health-related quality of life
IQ: Intelligence quotient
IMD: Inborn metabolic disease
K-ABC: Kaufman Assessment Battery for Children
PKU: Phenylketonuria
Total HrQoL: KINDL total HrQoL score
SDQ: Strengths and Difficulties Questionnaire
SSIEM: Society for the Study of Inborn Errors of Metabolism

## Code availability

N/A.
